# Supplementary material for: OpenMM 7: Rapid development of high performance algorithms for molecular dynamics
Source: PLoS Comput Biol. 2017 Jul 26;13(7):e1005659. doi: 10.1371/journal.pcbi.1005659 (PMC5549999; doi:10.1371/journal.pcbi.1005659)
Supplement: S1 Source Code — (ZIP) [file pcbi.1005659.s002.zip › OpenMM-7.0.1-Source/docs/api-python/generated/simtk.openmm.openmm.AmoebaMultipoleForce.html]

AmoebaMultipoleForce — OpenMM 7.0.1.dev-5e86c4f documentation


# AmoebaMultipoleForce¶

*class* `simtk.openmm.openmm.``AmoebaMultipoleForce`(*\*args*)¶
:   This class implements the Amoeba multipole interaction.

    To use it, create an AmoebaMultipoleForce object then call addMultipole() once for each atom. After an entry has been added, you can modify its force field parameters by calling setMultipoleParameters(). This will have no effect on Contexts that already exist unless you call updateParametersInContext().

    `__init__`(*self*) → AmoebaMultipoleForce¶
    :   \_\_init\_\_(self, other) -> AmoebaMultipoleForce

        Create an AmoebaMultipoleForce.

    Methods

    |  |  |
    | --- | --- |
    | `__init__`((self) -> AmoebaMultipoleForce) | \_\_init\_\_(self, other) -> AmoebaMultipoleForce |
    | `addMultipole`((self, charge, molecularDipole, ...) | Add multipole-related info for a particle |
    | `getAEwald`((self) -> double) | Get the Ewald alpha parameter. |
    | `getCovalentMap`(self, index, typeId) | Get the CovalentMap for an atom |
    | `getCovalentMaps`(self, index) | Get the CovalentMap for an atom |
    | `getCutoffDistance`((self) -> double) | Get the cutoff distance (in nm) being used for nonbonded interactions. |
    | `getElectrostaticPotential`(self, inputGrid, ...) | Get the electrostatic potential. |
    | `getEwaldErrorTolerance`((self) -> double) | Get the error tolerance for Ewald summation. |
    | `getExtrapolationCoefficients`((self) -> vectord) | Get the coefficients for the mu\_0, mu\_1, mu\_2, ..., mu\_n terms in the extrapolation algorithm for induced dipoles. |
    | `getForceGroup`((self) -> int) | Get the force group this Force belongs to. |
    | `getInducedDipoles`(self, context) | Get the induced dipole moments of all particles. |
    | `getMultipoleParameters`(self, index) | Get the multipole parameters for a particle. |
    | `getMutualInducedMaxIterations`((self) -> int) | Get the max number of iterations to be used in calculating the mutual induced dipoles |
    | `getMutualInducedTargetEpsilon`((self) -> double) | Get the target epsilon to be used to test for convergence of iterative method used in calculating the mutual induced dipoles |
    | `getNonbondedMethod`(...) | Get the method used for handling long-range nonbonded interactions. |
    | `getNumMultipoles`((self) -> int) | Get the number of particles in the potential function |
    | `getPMEParametersInContext`(self, context) | Get the parameters being used for PME in a particular Context. |
    | `getPmeBSplineOrder`((self) -> int) | Get the B-spline order to use for PME charge spreading |
    | `getPmeGridDimensions`(self) | Get the PME grid dimensions. |
    | `getPolarizationType`(...) | Get polarization type |
    | `getSystemMultipoleMoments`(self, context) | Get the system multipole moments. |
    | `setAEwald`(self, aewald) | Set the Ewald alpha parameter. |
    | `setCovalentMap`(self, index, typeId, ...) | Set the CovalentMap for an atom |
    | `setCutoffDistance`(self, distance) | Set the cutoff distance (in nm) being used for nonbonded interactions. |
    | `setEwaldErrorTolerance`(self, tol) | Get the error tolerance for Ewald summation. |
    | `setExtrapolationCoefficients`(self, coefficients) | Set the coefficients for the mu\_0, mu\_1, mu\_2, ..., mu\_n terms in the extrapolation algorithm for induced dipoles. |
    | `setForceGroup`(self, group) | Set the force group this Force belongs to. |
    | `setMultipoleParameters`(self, index, charge, ...) | Set the multipole parameters for a particle. |
    | `setMutualInducedMaxIterations`(self, ...) | Set the max number of iterations to be used in calculating the mutual induced dipoles |
    | `setMutualInducedTargetEpsilon`(self, ...) | Set the target epsilon to be used to test for convergence of iterative method used in calculating the mutual induced dipoles |
    | `setNonbondedMethod`(self, method) | Set the method used for handling long-range nonbonded interactions. |
    | `setPmeGridDimensions`(self, gridDimension) | Set the PME grid dimensions. |
    | `setPolarizationType`(self, type) | Set the polarization type |
    | `updateParametersInContext`(self, context) | Update the multipole parameters in a Context to match those stored in this Force object. |
    | `usesPeriodicBoundaryConditions`((self) -> bool) | Returns whether or not this force makes use of periodic boundary conditions. |

    Attributes

    |  |  |
    | --- | --- |
    | `Bisector` |  |
    | `Covalent12` |  |
    | `Covalent13` |  |
    | `Covalent14` |  |
    | `Covalent15` |  |
    | `CovalentEnd` |  |
    | `Direct` |  |
    | `Extrapolated` |  |
    | `LastAxisTypeIndex` |  |
    | `Mutual` |  |
    | `NoAxisType` |  |
    | `NoCutoff` |  |
    | `PME` |  |
    | `PolarizationCovalent11` |  |
    | `PolarizationCovalent12` |  |
    | `PolarizationCovalent13` |  |
    | `PolarizationCovalent14` |  |
    | `ThreeFold` |  |
    | `ZBisect` |  |
    | `ZOnly` |  |
    | `ZThenX` |  |

    `__delattr__`¶
    :   x.\_\_delattr\_\_(‘name’) <==> del x.name

    `__format__`()¶
    :   default object formatter

    `__getattribute__`¶
    :   x.\_\_getattribute\_\_(‘name’) <==> x.name

    `__hash__`¶

    `__reduce__`()¶
    :   helper for pickle

    `__reduce_ex__`()¶
    :   helper for pickle

    `__sizeof__`() → int¶
    :   size of object in memory, in bytes

    `__str__`¶

    `getForceGroup`(*self*) → int¶
    :   Get the force group this Force belongs to.

    `setForceGroup`(*self*, *group*)¶
    :   Set the force group this Force belongs to.

        |  |  |
        | --- | --- |
        | Parameters: | **group** (*int*) – the group index. Legal values are between 0 and 31 (inclusive). |

    `getNumMultipoles`(*self*) → int¶
    :   Get the number of particles in the potential function

    `getNonbondedMethod`(*self*) → OpenMM::AmoebaMultipoleForce::NonbondedMethod¶
    :   Get the method used for handling long-range nonbonded interactions.

    `setNonbondedMethod`(*self*, *method*)¶
    :   Set the method used for handling long-range nonbonded interactions.

    `getPolarizationType`(*self*) → OpenMM::AmoebaMultipoleForce::PolarizationType¶
    :   Get polarization type

    `setPolarizationType`(*self*, *type*)¶
    :   Set the polarization type

    `getCutoffDistance`(*self*) → double¶
    :   Get the cutoff distance (in nm) being used for nonbonded interactions. If the NonbondedMethod in use is NoCutoff, this value will have no effect.

        |  |  |
        | --- | --- |
        | Returns: | the cutoff distance, measured in nm |
        | Return type: | double |

    `setCutoffDistance`(*self*, *distance*)¶
    :   Set the cutoff distance (in nm) being used for nonbonded interactions. If the NonbondedMethod in use is NoCutoff, this value will have no effect.

        |  |  |
        | --- | --- |
        | Parameters: | **distance** (*double*) – the cutoff distance, measured in nm |

    `getAEwald`(*self*) → double¶
    :   Get the Ewald alpha parameter. If this is 0 (the default), a value is chosen automatically based on the Ewald error tolerance.

        |  |  |
        | --- | --- |
        | Returns: | the Ewald alpha parameter |
        | Return type: | double |

    `setAEwald`(*self*, *aewald*)¶
    :   Set the Ewald alpha parameter. If this is 0 (the default), a value is chosen automatically based on the Ewald error tolerance.

        |  |  |
        | --- | --- |
        | Parameters: | **aewald** (*double*) – alpha parameter |

    `getPmeBSplineOrder`(*self*) → int¶
    :   Get the B-spline order to use for PME charge spreading

        |  |  |
        | --- | --- |
        | Returns: | the B-spline order |
        | Return type: | int |

    `getPmeGridDimensions`(*self*)¶
    :   Get the PME grid dimensions. If Ewald alpha is 0 (the default), this is ignored and grid dimensions are chosen automatically based on the Ewald error tolerance.

        |  |  |
        | --- | --- |
        | Returns: | the PME grid dimensions |
        | Return type: | void |

    `setPmeGridDimensions`(*self*, *gridDimension*)¶
    :   Set the PME grid dimensions. If Ewald alpha is 0 (the default), this is ignored and grid dimensions are chosen automatically based on the Ewald error tolerance.

        |  |  |
        | --- | --- |
        | Parameters: | **gridDimension** (*vector< int >*) – the PME grid dimensions |

    `getPMEParametersInContext`(*self*, *context*)¶
    :   Get the parameters being used for PME in a particular Context. Because some platforms have restrictions on the allowed grid sizes, the values that are actually used may be slightly different from those specified with setPmeGridDimensions(), or the standard values calculated based on the Ewald error tolerance. See the manual for details.

        |  |  |
        | --- | --- |
        | Parameters: | **context** (*Context*) – the Context for which to get the parameters |
        | Returns: | - **alpha** (*double*) –   the separation parameter - **nx** (*int*) –   the number of grid points along the X axis - **ny** (*int*) –   the number of grid points along the Y axis - **nz** (*int*) –   the number of grid points along the Z axis |

    `addMultipole`(*self*, *charge*, *molecularDipole*, *molecularQuadrupole*, *axisType*, *multipoleAtomZ*, *multipoleAtomX*, *multipoleAtomY*, *thole*, *dampingFactor*, *polarity*) → int¶
    :   Add multipole-related info for a particle

        |  |  |
        | --- | --- |
        | Parameters: | - **charge** (*double*) – the particle’s charge - **molecularDipole** (*vector< double >*) – the particle’s molecular dipole (vector of size 3) - **molecularQuadrupole** (*vector< double >*) – the particle’s molecular quadrupole (vector of size 9) - **axisType** (*int*) – the particle’s axis type - **multipoleAtomZ** (*int*) – index of first atom used in constructing lab<->molecular frames - **multipoleAtomX** (*int*) – index of second atom used in constructing lab<->molecular frames - **multipoleAtomY** (*int*) – index of second atom used in constructing lab<->molecular frames - **thole** (*double*) – Thole parameter - **dampingFactor** (*double*) – dampingFactor parameter - **polarity** (*double*) – polarity parameter |
        | Returns: | the index of the particle that was added |
        | Return type: | int |

    `getMultipoleParameters`(*self*, *index*)¶
    :   Get the multipole parameters for a particle.

        |  |  |
        | --- | --- |
        | Parameters: | **index** (*int*) – the index of the atom for which to get parameters |
        | Returns: | - **charge** (*double*) –   the particle’s charge - **molecularDipole** (*vector< double >*) –   the particle’s molecular dipole (vector of size 3) - **molecularQuadrupole** (*vector< double >*) –   the particle’s molecular quadrupole (vector of size 9) - **axisType** (*int*) –   the particle’s axis type - **multipoleAtomZ** (*int*) –   index of first atom used in constructing lab<->molecular frames - **multipoleAtomX** (*int*) –   index of second atom used in constructing lab<->molecular frames - **multipoleAtomY** (*int*) –   index of second atom used in constructing lab<->molecular frames - **thole** (*double*) –   Thole parameter - **dampingFactor** (*double*) –   dampingFactor parameter - **polarity** (*double*) –   polarity parameter |

    `setMultipoleParameters`(*self*, *index*, *charge*, *molecularDipole*, *molecularQuadrupole*, *axisType*, *multipoleAtomZ*, *multipoleAtomX*, *multipoleAtomY*, *thole*, *dampingFactor*, *polarity*)¶
    :   Set the multipole parameters for a particle.

        |  |  |
        | --- | --- |
        | Parameters: | - **index** (*int*) – the index of the atom for which to set parameters - **charge** (*double*) – the particle’s charge - **molecularDipole** (*vector< double >*) – the particle’s molecular dipole (vector of size 3) - **molecularQuadrupole** (*vector< double >*) – the particle’s molecular quadrupole (vector of size 9) - **axisType** (*int*) – the particle’s axis type - **multipoleAtomZ** (*int*) – index of first atom used in constructing lab<->molecular frames - **multipoleAtomX** (*int*) – index of second atom used in constructing lab<->molecular frames - **multipoleAtomY** (*int*) – index of second atom used in constructing lab<->molecular frames - **thole** (*double*) – thole parameter - **dampingFactor** (*double*) – damping factor parameter - **polarity** (*double*) – polarity parameter |

    `setCovalentMap`(*self*, *index*, *typeId*, *covalentAtoms*)¶
    :   Set the CovalentMap for an atom

        |  |  |
        | --- | --- |
        | Parameters: | - **index** (*int*) – the index of the atom for which to set parameters - **typeId** (*CovalentType*) – CovalentTypes type - **covalentAtoms** (*vector< int >*) – vector of covalent atoms associated w/ the specfied CovalentType |

    `getCovalentMap`(*self*, *index*, *typeId*)¶
    :   Get the CovalentMap for an atom

        |  |  |
        | --- | --- |
        | Parameters: | - **index** (*int*) – the index of the atom for which to set parameters - **typeId** (*CovalentType*) – CovalentTypes type |
        | Returns: | **covalentAtoms** – output vector of covalent atoms associated w/ the specfied CovalentType |
        | Return type: | vector< int > |

    `getCovalentMaps`(*self*, *index*)¶
    :   Get the CovalentMap for an atom

        |  |  |
        | --- | --- |
        | Parameters: | **index** (*int*) – the index of the atom for which to set parameters |
        | Returns: | **covalentLists** – output vector of covalent lists of atoms |
        | Return type: | vector< std::vector< int > > |

    `getMutualInducedMaxIterations`(*self*) → int¶
    :   Get the max number of iterations to be used in calculating the mutual induced dipoles

        |  |  |
        | --- | --- |
        | Returns: | max number of iterations |
        | Return type: | int |

    `setMutualInducedMaxIterations`(*self*, *inputMutualInducedMaxIterations*)¶
    :   Set the max number of iterations to be used in calculating the mutual induced dipoles

        |  |  |
        | --- | --- |
        | Parameters: | **inputMutualInducedMaxIterations** (*int*) – number of iterations |

    `getMutualInducedTargetEpsilon`(*self*) → double¶
    :   Get the target epsilon to be used to test for convergence of iterative method used in calculating the mutual induced dipoles

        |  |  |
        | --- | --- |
        | Returns: | target epsilon |
        | Return type: | double |

    `setMutualInducedTargetEpsilon`(*self*, *inputMutualInducedTargetEpsilon*)¶
    :   Set the target epsilon to be used to test for convergence of iterative method used in calculating the mutual induced dipoles

        |  |  |
        | --- | --- |
        | Parameters: | **inputMutualInducedTargetEpsilon** (*double*) – target epsilon |

    `setExtrapolationCoefficients`(*self*, *coefficients*)¶
    :   Set the coefficients for the mu\_0, mu\_1, mu\_2, ..., mu\_n terms in the extrapolation algorithm for induced dipoles.

        |  |  |
        | --- | --- |
        | Parameters: | **coefficients** (*vector< double >*) – a vector whose mth entry specifies the coefficient for mu\_m. The length of this vector determines how many iterations are performed. |

    `getExtrapolationCoefficients`(*self*) → vectord¶
    :   Get the coefficients for the mu\_0, mu\_1, mu\_2, ..., mu\_n terms in the extrapolation algorithm for induced dipoles. In this release, the default values for the coefficients are [-0.154, 0.017, 0.658, 0.474], but be aware that those may change in a future release.

    `getEwaldErrorTolerance`(*self*) → double¶
    :   Get the error tolerance for Ewald summation. This corresponds to the fractional error in the forces which is acceptable. This value is used to select the grid dimensions and separation (alpha) parameter so that the average error level will be less than the tolerance. There is not a rigorous guarantee that all forces on all atoms will be less than the tolerance, however.

        This can be overridden by explicitly setting an alpha parameter and grid dimensions to use.

    `setEwaldErrorTolerance`(*self*, *tol*)¶
    :   Get the error tolerance for Ewald summation. This corresponds to the fractional error in the forces which is acceptable. This value is used to select the grid dimensions and separation (alpha) parameter so that the average error level will be less than the tolerance. There is not a rigorous guarantee that all forces on all atoms will be less than the tolerance, however.

        This can be overridden by explicitly setting an alpha parameter and grid dimensions to use.

    `getInducedDipoles`(*self*, *context*)¶
    :   Get the induced dipole moments of all particles.

        |  |  |
        | --- | --- |
        | Parameters: | **context** (*Context*) – the Context for which to get the induced dipoles |
        | Returns: | **dipoles** – the induced dipole moment of particle i is stored into the i’th element |
        | Return type: | vector< Vec3 > |

    `getElectrostaticPotential`(*self*, *inputGrid*, *context*)¶
    :   Get the electrostatic potential.

        |  |  |
        | --- | --- |
        | Parameters: | - **inputGrid** (*vector< Vec3 >*) – input grid points over which the potential is to be evaluated - **context** (*Context*) – context |
        | Returns: | **outputElectrostaticPotential** – output potential |
        | Return type: | vector< double > |

    `getSystemMultipoleMoments`(*self*, *context*)¶
    :   Get the system multipole moments.

        This method is most useful for non-periodic systems. When called for a periodic system, only the <i>lowest nonvanishing moment</i> has a well defined value. This means that if the system has a net nonzero charge, the dipole and quadrupole moments are not well defined and should be ignored. If the net charge is zero, the dipole moment is well defined (and really represents a dipole density), but the quadrupole moment is still undefined and should be ignored.

        |  |  |
        | --- | --- |
        | Parameters: | **context** (*Context*) – context |
        | Returns: | **outputMultipoleMoments** – (charge, dipole\_x, dipole\_y, dipole\_z, quadrupole\_xx, quadrupole\_xy, quadrupole\_xz, quadrupole\_yx, quadrupole\_yy, quadrupole\_yz, quadrupole\_zx, quadrupole\_zy, quadrupole\_zz) |
        | Return type: | vector< double > |

    `updateParametersInContext`(*self*, *context*)¶
    :   Update the multipole parameters in a Context to match those stored in this Force object. This method provides an efficient method to update certain parameters in an existing Context without needing to reinitialize it. Simply call setMultipoleParameters() to modify this object’s parameters, then call updateParametersInContext() to copy them over to the Context.

        This method has several limitations. The only information it updates is the parameters of multipoles. All other aspects of the Force (the nonbonded method, the cutoff distance, etc.) are unaffected and can only be changed by reinitializing the Context. Furthermore, this method cannot be used to add new multipoles, only to change the parameters of existing ones.

    `usesPeriodicBoundaryConditions`(*self*) → bool¶
    :   Returns whether or not this force makes use of periodic boundary conditions.

        |  |  |
        | --- | --- |
        | Returns: | true if nonbondedMethod uses PBC and false otherwise |
        | Return type: | bool |

High performance molecular simulation on GPUs


### Navigation

- Application Layer
- Library Layer
  - Core Objects
  - Forces
  - Integrators
  - Extras

©2015, Stanford University and the Authors.
|
Powered by Sphinx 1.3.1
& Alabaster 0.7.7
|
Page source
